# Activation of IL1 signaling molecules by Kaposi’s sarcoma-associated herpesvirus

**DOI:** 10.3389/fcimb.2022.1049624

**Published:** 2022-11-15

**Authors:** Jungang Chen, Jiao Song, Jennifer James, Karlie Plaisance-Bonstaff, Steven R. Post, Zhiqiang Qin, Lu Dai

**Affiliations:** ^1^Department of Pathology, Winthrop P. Rockefeller Cancer Institute, University of Arkansas for Medical Sciences, Little Rock, AR, United States; ^2^Department of Medicine, Louisiana State University Health Sciences Center, Louisiana Cancer Research Center, New Orleans, LA, United States

**Keywords:** Kaposi’s Sarcoma, PEL, IL1, KSHV, IL1RAP

## Abstract

**Objective:**

Kaposi’s Sarcoma-associated Herpesvirus (KSHV) is the etiologic agent of several human cancers, including Kaposi’s sarcoma (KS) and Primary effusion lymphoma (PEL), which are usually seen in immunocompromised patients while lack of effective therapeutic options. Interleukin1 (IL1) family is a major mediator for inflammation response and has functional role in both innate and adaptive immunity. In contrast to the well-studied IL1 molecules, the activation and functional role of IL1 receptor/co-receptor and other related ligands, such as the IL1 receptor accessory protein (IL1RAP), in KSHV pathogenesis and tumorigenesis remain almost unknown.

**Methods:**

In the current study, a series of KSHV negative and positive primary or tumor cells, as well as AIDS-KS tumor samples from cohort HIV+ patients were used to compare and determine the activation status of IL1 signaling molecules, and their functional roles in KSHV pathogenesis.

**Results:**

We reported the high activation of multiple IL1 signaling molecules, including IL1, IL36, IL1R1, IL1RAP and IRAKs, during KSHV latent and lytic stages, as well as in clinical samples from patients with KSHV-related malignancies. Directly targeting these molecules especially IL1R1 and IL1RAP significantly impaired the survival and growth of KSHV+ tumor cells, as well as their colony formation on 3-D culture.

**Conclusion:**

Our data indicate the importance of IL1 signaling molecules in KSHV pathogenesis and tumorigenesis, which may represent attractive therapeutic targets against these virus-associated diseases.

## Introduction

Kaposi’s sarcoma-associated herpesvirus (KSHV) can cause several cancers which are usually seen in immunocompromised patients, especially Kaposi’s Sarcoma (KS) and Primary Effusion Lymphoma (PEL) (Naimo et al., 2021). Despite the reduced incidence of KS in the era of combined Antiretroviral Therapy (cART) developed for Human Immunodeficiency Virus (HIV)-infected patients, KS still remains one of common Acquired Immunodeficiency Syndrome (AIDS)-associated tumors (especially in some Africa countries) (Bonnet et al., 2004; Engels et al., 2008). Another KSHV-related malignancy, PEL, is a rapidly progressing B cell-derived lymphoma with poor prognosis, even under the combinational chemotherapy (Chen et al., 2007). PEL comprises transformed B cells which carrying KSHV episome and is mostly found within the pleural or peritoneal cavities of immunosuppressed patients such as HIV-infected individuals.

Interleukin1 (IL1) is an inflammatory cytokine family, which contains 11 distinct proteins and has a wide array of functions in host innate immunity. The IL1 superfamily contains many pro-inflammatory cytokines such as IL1α, IL1β, IL18, IL33, IL36 and a few anti-inflammatory cytokines (Boraschi and Tagliabue, 2013). Among them, IL1 is the defining member of this family, so its physiological and pathological functions have been thoroughly studied and reported. IL1 includes two activator cytokines, IL1α and IL1β. IL1 signaling activation can be through canonical or non-canonical NLRP3 inflammasomes. Canonical activation occurs after the sensing of factors by NLRP3, causing the assembly of NLRP3, ASC and caspase-1 into a complex that results in the activation of caspase-1 mediated proteolytic processing of pro-IL1 proteins such as pro-IL1β and pro-IL18 (Afonina et al., 2015; Pyrillou et al., 2020). Alternatively, non-canonical pathways of NLRP3 inflammasome activation are associated with several pro-inflammatory caspases, such as caspase-4, caspase-5, and caspase-11. In this process, bacterial products especially LPS are recognized by the caspase-recruitment domain (CARD) of respective caspases, leading to IL1 activation (Kayagaki et al., 2013; Shi et al., 2014). Upon stimulation, IL1 binds to the type I IL1 receptor (IL1R1) which then recruits the IL1 receptor accessory protein (IL1RAP), the adaptor protein MyD88, as well as IL-1R-associated kinases such as IRAK1, IRAK2, and IRAK4. Once the IL1 receptor complex is formed, they are able to activate a downstream signaling cascade which stimulates different immune responses and inflammatory genes (Jensen, 2017). A lot of studies suggest that inflammatory and angiogenic cytokines including IL1 contribute to KS pathogenesis through inducing abnormal proliferation, angiogenesis, as well as a KS-like phenotype (Ensoli et al., 1992). For example, IL1β is markedly elevated in most KS lesions (Samaniego et al., 1997). Several studies have found that KSHV infection and some KSHV-encoded proteins are able to induce IL1α & IL1β production from host cells (Singh et al., 2013; Host et al., 2017). Not surprisingly, the expressional levels of many IL1 target genes such as CCL2, IL6 and IL8 are all elevated in KSHV-infected cells or KSHV+ tumor tissues (Ensoli et al., 1992). We recently report strong PD-1/PD-L1/PD-L2 expression in AIDS-KS tissues, and that KSHV lytic reactivation further induces PD-L1 expression from tumor cells through IL1β (Chen et al., 2019). In the current study, we have investigated the activated status of IL1 signaling molecules during KSHV infection (including both latent and lytic phases) and in clinical samples from HIV+ patients with KSHV-associated malignancies.

## Materials and methods

### Cell culture, reagents and infection protocols

KSHV+ PEL cell line BCBL-1 was kindly gifted by Dr. Dean Kedes (University of Virginia), cultured in RPMI 1640 media with supplemented with 10% fetal bovine serum (FBS), 10 mM HEPES, 100 U/mL penicillin, 100 µg/mL streptomycin, 2 mM L-glutamine, 0.05 mM β-mercaptoethanol, and 0.02% (wt/vol) sodium bicarbonate. Primary human umbilical vein endothelial cells (HUVEC) and BL-41 cell line were purchased from American Type Culture Collection (ATCC), and cultured as recommended by ATCC. The TREx BCBL1-RTA cell line was kindly gifted by Dr. Pinghui Feng (University of Southern California). For KSHV infection experiments, BCBL-1 cells were incubated with 0.6 mM valproic acid for 4-6 days, then the virions was purified from the culture supernatant by using the ultracentrifugation at 20,000 × g for 3 h, 4°C. The viral pellets were resuspended in 1/100 of the original volume with cell culture medium. The infectious titers of virus were determined as described previously (Chen et al., 2019).

### RT-qPCR

Total RNA was isolated by using the RNeasy Mini kit (Qiagen), and cDNA was synthesized using a SuperScript III First-Strand Synthesis SuperMix Kit (Invitrogen). Specific primers used for amplification of individual target gene have been listed in **Table S1**. The amplification was carried out using an iCycler IQ Real-Time PCR Detection System, and cycle threshold (Ct) values were tabulated in triplicate for each gene of interest in each experiment. “No template” (water) controls were used to ensure minimal background contamination. Using mean Ct values tabulated for each gene, and the paired Ct values for *β-actin* gene as a loading control, the fold changes for experimental groups relative to assigned control groups were calculated by using automated iQ5 2.0 software (Bio-rad).

### RNA interference

For RNAi assays, IL1RAP or IL1R1 On-Target plus SMARTpool small interfering RNA (siRNA; Dharmacon) or negative control siRNA were delivered by using the DharmaFECT transfection reagents, as recommended by the manufacturer.

### Cell proliferation and apoptosis assays

Cell proliferation was measured using the WST-1 Colorimetric Assay (Roche). Briefly, after the treatments, 10 μL/well of cell proliferation detection reagent, WST-1 (4-[3-(4-Iodophenyl)-2-(4-nitro- phenyl)-2H-5-tetrazolio]-1,3-benzene disulfonate), was added into 96-well microplate and incubated for additional 3 h at 37°C, 5% CO_2_ conditions. The absorbance of samples was then measured by using a microplate reader at 450 nm. The flow cytometry was used for the quantitative assessment of apoptosis with the fluorescein isothiocyanate–Annexin V/propidium iodide (PI) Apoptosis Detection Kit I (BD Pharmingen) and analyzed on a FACS Calibur 4-color flow cytometer (BD Bioscience).

### Western blot

Total cell lysates were resolved by 10% SDS–PAGE, transferred to nitrocellulose membranes, and immunoblotted with antibodies for viral protein LANA (Advanced Biotechnologies) and RTA (ABBIOTEC), IL1R1, IL1RAP (Abcam), cleaved Caspase 3/9, cleaved PARP, and Tubulin as a loading control (Cell Signaling). Immunoreactive bands were identified by using an enhanced chemiluminescence reaction (Perkin-Elmer), then visualized by autoradiography.

### PEL 3-D culture

To generate a high density of microcolonies in a scaffold-free model, BCBL-1 cells were transfected with siRNAs as described above for 24 h, then seeded into the Coring Elplasia 96-well round bottom microplate featuring Ultra-Low Attachment (ULA) surface, ~2,000 cells/well (Corning). After 72 h culture, the colony formation was detected and measured using a microscope.

### AIDS-KS tumor tissues and immunohistochemistry

KS tissues from patients with HIV infection were provided by the Louisiana State University Health Sciences Center (LSUHSC) HIV Outpatient (HOP) Clinic and Biospecimens Bank. The study was approved by the Institutional Review Board for Human Research (approval no. 8079) at LSUHSC. All subjects have been provided with written informed consent. Immunohistochemistry was performed as described previously (Dai et al., 2018). The antibody for viral protein LANA was purchased from Advanced Biotechnologies. All the other antibodies for IL1α, IL1R1, IL1RAP, IL36α, IL36γ, IRAK1, IRAK2 and IRAK4 were purchased from Abcam and used as recommended by the manufacturer. The images were collected by using an Olympus BX61 microscope equipped with a high resolution DP72 camera and CellSense image capture software.

### Statistical analysis

Significance for differences between experimental and control groups (with no fewer than 3 experiments per group performed) was determined using the two-tailed Student’s t-test (Excel 2016), and p values < 0.05 or < 0.01 were considered significant or highly significant, respectively.

## Results

### Activation of IL1 signaling molecules during KSHV infection

**Figure 1:**
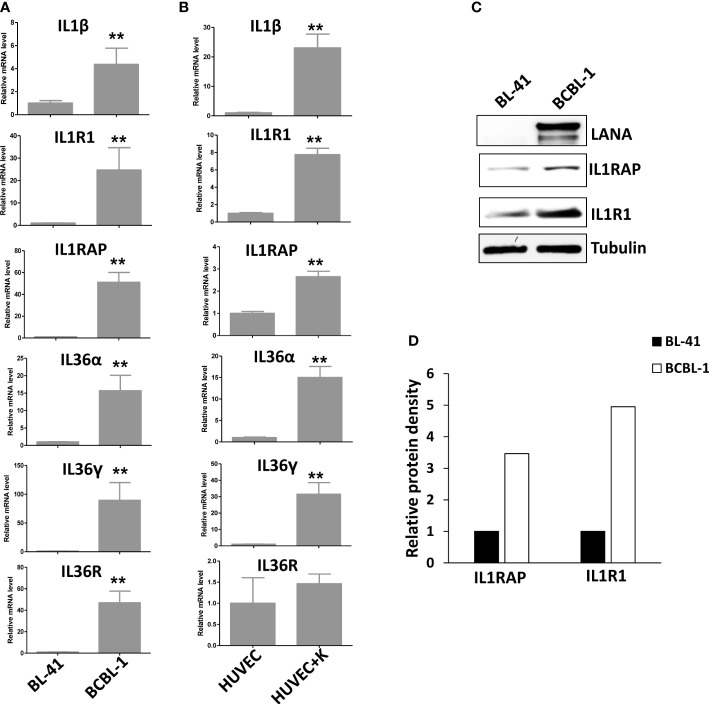
Activation of IL1 signaling in KSHV-infected PEL and primary endothelial cells. **(A)** Gene expression was detected and compared between BL-41 (a KSHV negative lymphoma cell line) and BCBL-1 (a KSHV+ PEL cell line) by using RT-qPCR. **(B)** Primary endothelial cells HUVEC were infected with KSHV (MOI~5) or not for 48 h, followed by RT-qPCR analysis. Error bars represent the S.D. for 3 independent experiments. **p<0.01. **(C, D)** The protein expression was measured using Western blot, and representative blots from one of two independent experiments were shown. The density of protein bands was scanned and quantified using Image J software.

**Figure 2:**
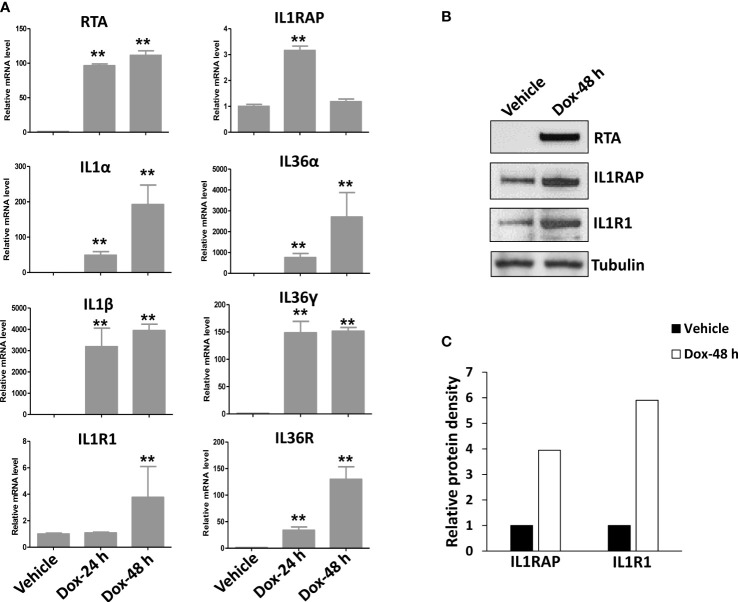
Further activation of IL1 signaling during KSHV lytic reactivation. The TREx BCBL1-Rta cell line was induced to lytic reactivation after exposure to 1 μg/mL of doxycycline (Dox) for 24 or 48 h, respectively. **(A)** Gene expression was then detected and compared by using RT-qPCR. RTA represents a key viral gene controlling KSHV “latency to lytic” switch. Error bars represent the S.D. for 3 independent experiments. **p<0.01. **(B, C)** The protein expression was measured using Western blot, and representative blots from one of two independent experiments were shown. The density of protein bands was scanned and quantified using Image J software.

After screening of the major ligands and receptors/co-receptors in the IL1 signaling, we found that compared to BL-41, a KSHV negative lymphoma cell line, KSHV+ PEL cell lines such as BCBL-1 displayed elevated levels of many IL1 signaling molecules, some of which having dramatically increasing, especially IL1β, IL1R1, IL1RAP, IL36α, IL36γ and IL36R, while the others not changed or undetectable such as IL1α, IL33, using RT-qPCR (**Figure 1A**). We also found that KSHV *de novo* infection of primary endothelial cells increased the levels of IL1 signaling molecules, such as IL1β, IL1R1, IL1RAP, IL36α and IL36γ (**Figure 1B**). Western blot results further confirmed the upregulation of IL1 signaling molecules such as IL1RAP and IL1R1 expression in KSHV infected cells (**Figures 1C, D**). Interestingly, we observed that induction of KSHV lytic reactivation from the inducible TREx BCBL1-RTA cell line (which was confirmed by the upregulation of viral RTA expression induced by Dox) further activated IL1 signaling molecules, including IL1α, IL1β, IL1R1, IL1RAP, IL36α, IL36γ and IL36R (**Figures 2A–C**). Together, these data demonstrate the activation of IL1 signaling molecules in both KSHV latent and lytic phases, indicating the regulation of IL1 signaling during the whole viral life cycle.

### Targeting IL1RAP and IL1R1 inhibits the growth of BCBL-1 PEL cells

**Figure 3:**
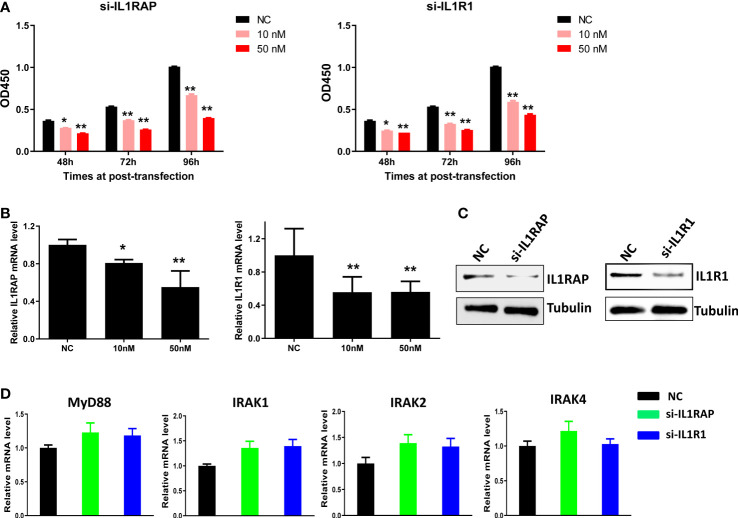
Targeting IL1RAP and IL1R1 by RNAi inhibits the growth of BCBL-1 PEL cells. **(A)** BCBL-1 were transfected with 10 nM or 50 nM of IL1RAP-siRNA, IL1R1-siRNA or non-target control siRNA (NC at 50 nM) (Dharmacon SMARTpool siRNA) for 48-96 h, then the cell proliferation was measured using the WST-1 assays. **(B–D)** Cells were transfected as above for 72 h, then the target genes transcription was measured using RT-qPCR. The protein expression was measured using Western blot, and representative blots from one of two independent experiments were shown. Error bars represent S.D. for 3 independent experiments, *p<0.05, **p<0.01.

Since we found that IL1 signaling receptors/co-receptors such as IL1R1 and IL1RAP were highly expressed in KSHV+ PEL cells, we used specific siRNA to directly silence them from BCBL-1 cell line. We then found that knockdown of either gene significantly reduced the growth of BCBL-1 lymphoma cell in a dose-dependent manner (**Figures 3A–C**). We also examined the impacts of knockdown of IL1R1 or IL1RAP on other IL1 signaling molecules. We found out knockdown of either IL1R1 or IL1RAP almost not affecting the expression of MyD88, IRAK1, IRAK2 and IRAK4 (**Figure 3D**), implying these molecules are not directly regulated by IL1R1 or IL1RAP.

**Figure 4:**
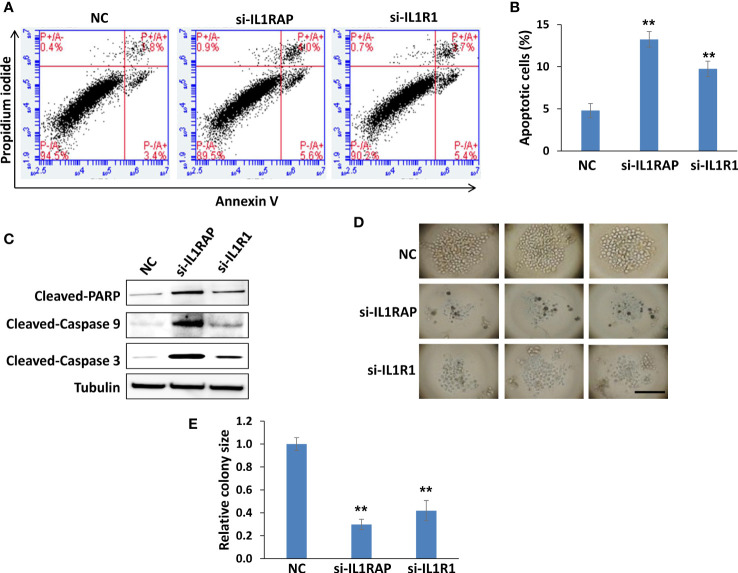
Targeting IL1RAP and IL1R1 by RNAi induces PEL cell apoptosis and impairs PEL colony formation. **(A, B)** BCBL-1 were transfected with 50 nM of IL1RAP-siRNA, IL1R1-siRNA or non-target control siRNA (NC) (Dharmacon SMARTpool siRNA) for 48 h, then the cell apoptosis was quantified using flow cytometry analysis. Error bars represent S.D. for 3 independent experiments, ** = p<0.01. **(C)** The protein expression was measured using Western blot, and representative blots from one of two independent experiments were shown. **(D, E)** BCBL-1 were transfected as described above, then seeded into the Coring Elplasia 96-well round bottom microplate featuring Ultra-Low Attachment (ULA) surface. After 72 h culture, the colony formation was detected and measured using a microscope. Error bars represent S.D. for 6 wells from each group, **p<0.01. Bar: 100 μm.

Using flow cytometry, we found that knockdown of either gene significantly induced PEL cell apoptosis (**Figures 4A, B**), which was further confirmed by the increased cleavage of PARP, Caspases-3 and -9, these major apoptosis markers from IL1R1 or IL1RAP knockdown lymphoma cells (**Figure 4C**). We next used a 3-D scaffold-free model to assess the effects of targeting IL1RAP and IL1R1 on colony formation ability of PEL cells. As shown in **Figures 4D, E**, direct knockdown of either IL1R1 or IL1RAP significantly impaired colony formation of BCBL-1 cells. Moreover, in those colonies formed from gene knockdown groups, there were many dead or unhealthy cells, which were not seen in those from the control group. These data together indicate that both L1RAP and IL1R1 may contribute to KSHV+ tumor cell survival or proliferation, which may represent potential therapeutic targets.

### Upregulation of IL1 signaling molecules within AIDS-KS tissues

**Figure 5:**
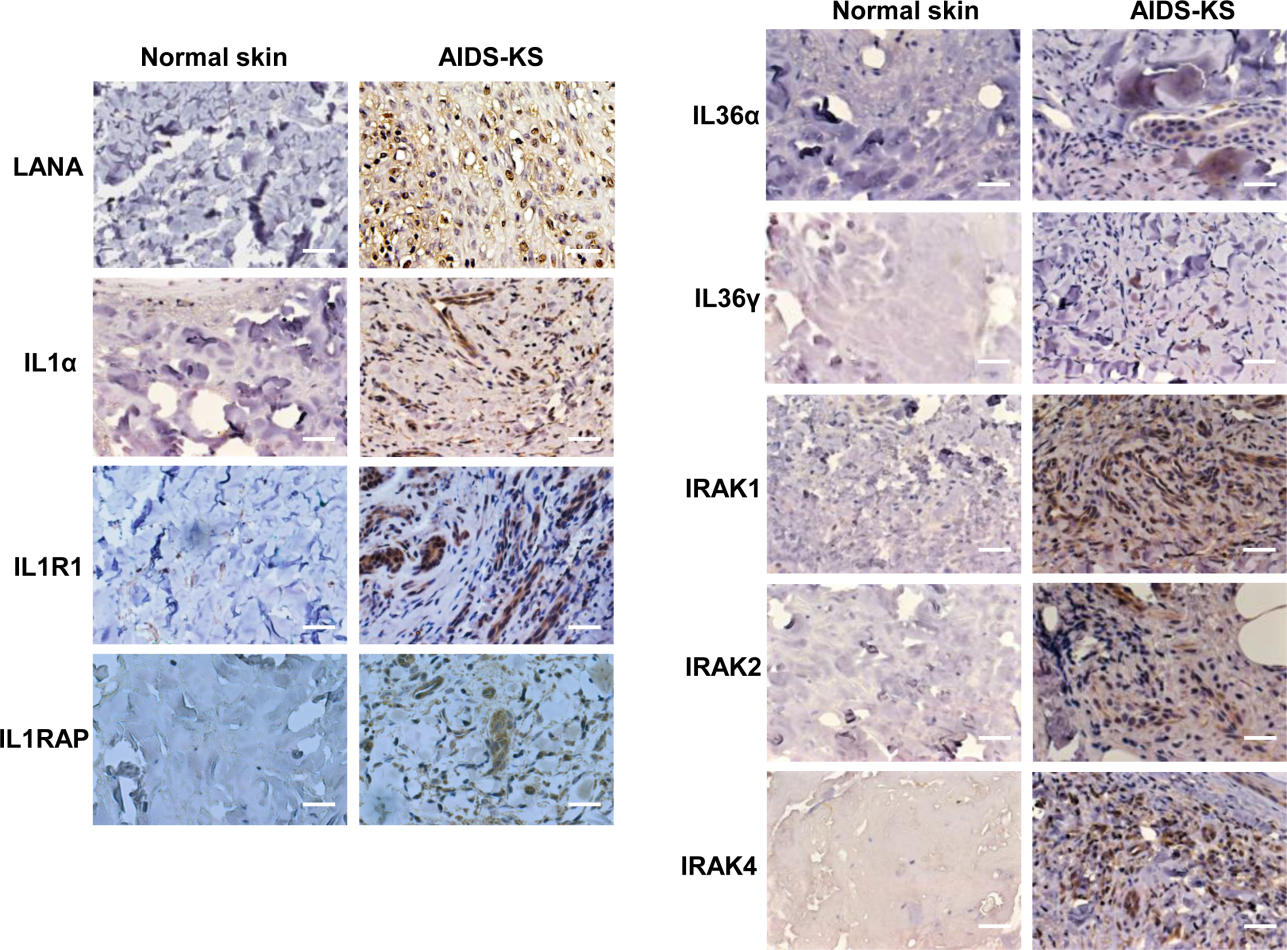
The upregulation of IL1 signaling molecules within AIDS-KS tissues. Expression of viral latent protein LANA and host IL1 signaling molecules in formalin-fixed paraffin-embedded KS tissues from cohort HIV+ patient and normal skin tissues were determined and compared by using immunohistochemical staining as described in the Methods (the magnification at x40). Bars: 50 μm.

For clinical implication, we compared the expression of IL1 signaling molecules between normal skin and AIDS-KS tissues using IHC staining. First, the abundant expression of viral latent protein, LANA (the marker of KSHV latent infection), was only found in AIDS-KS tissues but not in normal skin tissue. Next, our results showed that the expression of several IL1 signaling molecules including IL1α, IL1R1, IL1RAP, IL36α, and IL36γ were indeed upregulated in AIDS-KS tissues when compared to those in normal skin tissues (**Figure 5**). Interestingly, the mutation of IRAK1 has been reported in KSHV+ PEL cells and related to PEL survival (Yang et al., 2014). Thus, we also examined the expression of different IRAK isoforms in clinical samples. We found that all of 3 IRAK isoforms (IRAK1, IRAK2, and IRAK4) we tested were highly expressed in AIDS-KS tissues when compared to those in normal skin tissues (**Figure 5**).

## Discussion

In the current study, we found that many of IL1 signaling molecules (e.g., IL1α, IL1β, IL1R1, IL1RAP, IL36, IL36R and IRAKs) were highly activated during KSHV infection or in KSHV+ tumor cells and tissues, indicating the crucial role of these molecules functions in KSHV pathogenesis and oncogenesis. In contrast to the well-studied IL1, many other molecules especially IL1RAP, its functions in infectious disease and cancer including KSHV infection and related diseases remain largely unclear. The importance of co-receptor IL1RAP in the signaling of the IL1 family of alarmins has been confirmed by genetic deletion of IL1RAP that led to a complete loss of signaling for IL1, IL33 and IL36 (Boraschi and Tagliabue, 2013), indicating IL1RAP as a global regulator in the IL1 signaling and that targeting IL1RAP may have a broad anti-IL1 activity as the advantage. Interestingly, IL1RAP has been found frequently expressed in different leukemic stem cells (LSCs) (Blatt et al., 2018; Eisenwort et al., 2019), connecting its functions to tumorigenesis. Our current study also reported the upregulation of IL1RAP in AIDS-KS tissues when compared to those normal skin tissues. Interestingly, IL33, another IL1 family member, has been suggested probably involved in KSHV pathogenesis by regulation of chromatin compaction through the nucleosome-nucleosome interactions (Roussel et al., 2008). However, our study detected very low level of IL33 or IL33R expression in KSHV-infected cells (data not shown).

The inflammasome contains different innate immune system receptors and sensors that can regulate the activation of caspase-1 and induce the inflammation in response to a lot of infectious microbes and host proteins (Guo et al., 2015). Inflammasome formation results in the proteolytic processing of the pro-inflammatory cytokines IL1β and IL18 by active caspase-1, which is associated with pyroptosis, an inflammatory process involving caspase-1 mediated cell death. Interestingly, several recent studies have revealed KSHV infection regulation of inflammasome in host cells. For example, KSHV Orf63 is a viral protein homologous to human NLRP1, and able to block NLRP1-dependent innate immune responses (Gregory et al., 2011). Also, during KSHV infection of endothelial cells, interferon gamma-inducible protein 16 (IFI16) can interact with the adaptor molecules, ASC and procaspase-1, to together form a functional inflammasome (Kerur et al., 2011; Ansari et al., 2015). However, the remained question is whether and how other IL1 signaling molecules (e.g., IL1R1, IL1RAP, IL36) are potentially connected to inflammasome formation and functions within KSHV-infected cells, which require further investigation.

Our data showed that directly silencing of IL1 signaling molecules such as IL1R1 and IL1RAP significantly reduced the survival and growth of KSHV+ tumor cells, indicating targeting IL1 signaling may represent a promising strategy for treatment of KSHV-related malignancies. In fact, currently there are three major categories of IL1 blockers approved by the US Food and Drug Administration (FDA) for clinical usage: 1) The IL1 receptor antagonist (e.g., Anakinra), which can block the IL1 receptor and reduce the activities of IL1α and IL1β; 2) The soluble decoy receptor (e.g., Rilonacept, also known as IL1 Trap), a dimeric fusion protein consisting of the ligand-binding domains of IL1R1 and IL1RAP linked in-line to the fragment-crystallizable portion (Fc region) of human IgG1 that can bind and neutralize IL1; 3) The neutralizing monoclonal anti-IL1β antibody (e.g., Canakinumab), which can directly target IL1β (Dinarello, 2013; Dinarello and van der Meer, 2013). In addition, it is speculated that targeting some unique epitope of IL1RAP protein may result in blocking IL1, IL33, and IL36 signaling simultaneously. However, most of them have not been tried in KSHV-related malignancies models for their efficacy.

In summary, our study demonstrate the high activation of multiple IL1 signaling molecules during KSHV infection including both latent and lytic phases, as well as in clinical samples from patients with KSHV-related malignancies. These data indicate the importance of IL1 signaling molecules in KSHV pathogenesis and oncogenesis, which may represent attractive therapeutic targets against these diseases.

## Data availability statement

The raw data supporting the conclusions of this article will be made available by the authors, without undue reservation.

## Ethics statement

The studies involving human participants were reviewed and approved by KS tissues from HIV-infected patients were provided by the Louisiana State University Health Sciences Center (LSUHSC) HIV Outpatient (HOP) Clinic and Biospecimens Bank. The study was approved by the Institutional Review Board for Human Research (approval no. 8079) at LSUHSC. All subjects provided written informed consent. The patients/participants provided their written informed consent to participate in this study.

## Author contributions

JC, ZQ, and LD designed and performed experiments, analyzed results, wrote the manuscript. JS, JJ, and KP-B, performed experiments. SP edited the manuscript and provided critical input. All authors contributed to the article and approved the submitted version.

## Funding

This work was supported by NIH R01CA228166, R03DE031978, the Arkansas Bioscience Institute, the major research component of the Arkansas Tobacco Settlement Proceeds Act of 2000. Part of this work was also supported by the Winthrop P. Rockefeller Cancer Institute Core Facility Voucher Award, and VCRI Pioneer Award. Funding sources had no role in study design, data collection and analysis, decision to publish, or preparation of the manuscript.

## Conflict of interest

The authors declare that the research was conducted in the absence of any commercial or financial relationships that could be construed as a potential conflict of interest.

## Publisher’s note

All claims expressed in this article are solely those of the authors and do not necessarily represent those of their affiliated organizations, or those of the publisher, the editors and the reviewers. Any product that may be evaluated in this article, or claim that may be made by its manufacturer, is not guaranteed or endorsed by the publisher.
