# Supplementary material for: Activation of IL1 signaling molecules by Kaposi’s sarcoma-associated herpesvirus
Source: Front Cell Infect Microbiol. 2022 Nov 15;12:1049624. doi: 10.3389/fcimb.2022.1049624 (PMC9705745; doi:10.3389/fcimb.2022.1049624)
Supplement: Supplementary file 1 [file Table_1.docx]

**Supplemental Table 1. Primer sequences for RT-qPCR.**

| **Gene** | **Sequences (5’ 3’)** |
| --- | --- |
| *IL1α* | *sense CGCCCTCAATCAAAGTAT*  *antisense TGGGTATCTCAGGCATCT* |
| *IL1β* | *sense ACAGTGGCAATGAGGATG*  *antisense TGTAGTGGTGGTCGGAGA* |
| *IL1R1* | *sense CTGTCCTCTTAACCCAAAT*  *antisense CAAGTCCTCCGTCTCCTG* |
| *IL1RAP* | *sense GACTGTAAAGGTAGTAGGCTCT*  *antisense ATCAATGGTCCACCAAAC* |
| *IL33* | *sense TGGAGGATGAAAGTTATG*  *antisense TTGTAGGACTCAGGGTTA* |
| *IL33R* | *sense ACAACTGGACAGCACCTC*  *antisense CTCCGATTACTGGAAACA* |
| *IL36α* | *sense CAGGACCAGACGCTCATA*  *antisense ATTCAGGCCCAGGTAGAT* |
| *IL36β* | *sense GCCTGTCACTCTTCATTT*  *antisense TCTACCCACTCCTATTCC* |
| *IL36γ* | *sense GGGCCGTCTATCAATCAA*  *antisense GCAATGTGGGCTGTTCTC* |
| *IL36R* | *sense CTTACTTGATAGGAGGGC*  *antisense TGTAGAATGGAAGGCACT* |
| *IRAK1* | *sense CCTGGATCAACCGCAACG*  *antisense GGTCTGGGAGCCTGGAAAA* |
| *IRAK2* | *sense ATATTCCAAGCAGCACCG*  *antisense TCAGAAAGCCCACTCCAA* |
| *IRAK4* | *sense ATATGCCACCTGACTCCT*  *antisense AACTCCAAATCCTCCCTC* |
| *β-actin* | *sense GGAAATCGTGCGTGACATT*  *antisense GACTCGTCATACTCCTGCTTG* |
